# Exploring the impact of a co‐created dementia awareness session on dementia knowledge and attitudes in culturally diverse carers: a cross‐sectional analysis

**DOI:** 10.1002/alz.095185

**Published:** 2025-01-09

**Authors:** Manjari Manimaran, Gabriela E Caballero, Eman Shatnawi, Emy Tsouros, Mai Tran, Nibras Jasim, An Nguyen, Emily Cope, Sonia Chan, Joyce Siette, Ann Dadich, Michelle DiGiacomo, Genevieve Z Steiner‐Lim, Diana Karamacoska

**Affiliations:** ^1^ Western Sydney University, Sydney, NSW Australia; ^2^ NICM Health Research Institute, Western Sydney University, Sydney, NSW Australia; ^3^ MARCS Institute for Brain, Behaviour and Development, Western Sydney University, Penrith, NSW Australia; ^4^ University of Technology Sydney, Sydney, NSW Australia; ^5^ Translational Health Research Institute, Western Sydney University, Penrith, NSW Australia

## Abstract

**Background:**

Many carers of people living with dementia display misperceptions and negative attitudes about dementia due to limited education. This stigma can impact care and the accessibility of appropriate support services. Culturally diverse people living with dementia and their carers remain underserviced and lack culturally inclusive resources despite having specialised dementia needs. Our study aimed to explore the impact of a co‐created dementia awareness‐raising session on the dementia knowledge and attitudes of culturally and linguistically diverse carers.

**Method:**

A quasi‐experimental cross‐sectional study was conducted in South‐Western Sydney, Australia, targeting English, Arabic, Vietnamese, Cantonese, Mandarin, and Greek‐speaking carers. A survey comprising 10 items from the dementia knowledge assessment scale (DKAS) and 15 items from the dementia attitudes scale (DAS) was administered to carers who attended a two‐hour awareness‐raising session co‐created with community stakeholders (intervention group) and online to carers who self‐reported having no previous dementia education (comparison group). The surveys and information sessions were translated and conducted in‐language by bilingual educators. Mann‐Whitney U tests were used to compare the mean rank differences between the two groups on each scale item.

**Results:**

For 8 of 10 DKAS items that assessed knowledge of the risk factors, presentation, and management of dementia, the intervention group (n = 42) had a higher proportion of correct answers relative to the comparison group (n = 74) (all *p* ≤.041). For 3 of 15 DAS items, regarding self‐perceived knowledge and awareness of local services, the intervention group (n = 188) displayed more positive attitudes relative to the comparison group (n = 427) (all *p* <.001).

**Conclusion:**

Our co‐created multilingual dementia education initiative was effective in supporting dementia knowledge and awareness of local services among carers. However, the single session was likely insufficient to modify attitudes towards people living with dementia. The observational design used limits our understanding of baseline levels of knowledge and attitudes and thus the impact of this initiative. Future pre/post‐test experimental research should examine the improvement of dementia knowledge and attitudes with longer‐term carer‐specific education.

